# Implementation of Food Safety Management Systems along with Other Management Tools (HAZOP, FMEA, Ishikawa, Pareto). The Case Study of *Listeria monocytogenes* and Correlation with Microbiological Criteria

**DOI:** 10.3390/foods10092169

**Published:** 2021-09-13

**Authors:** Jocelyn C. Lee, Aura Daraba, Chrysa Voidarou, Georgios Rozos, Hesham A. El Enshasy, Theodoros Varzakas

**Affiliations:** 1 Food Safety Consultant, 836B Southampton Road, Suite 355, Benicia, CA 94510, USA; jlee@gourmetrail.com; 2 Faculty of Food Science and Engineering, “Dunarea de Jos” University of Galati, 111 Domneasca Street, Build. F, 800201 Galati, Romania; Aura.Daraba@ugar.ro; 3 Laboratory of Animal Health, Food Hygiene and Quality, Department of Agriculture, University of Ioannina, GR47100 Arta, Greece; xvoidarou@uoi.gr (C.V.); clevervet@hotmail.com (G.R.); 4 Institute of Bioproduct Development (IBD), Universiti Teknologi Malaysia (UTM), Johor Bahru 81310, Malaysia; henshasy@ibd.utm.my; 5 City of Scientific Research and Technology Applications (SRTA), New Burge Al Arab, Alexandria 21934, Egypt; 6 Food Science and Technology, University of Peloponnese, 24100 Kalamata, Greece

**Keywords:** food safety, Prerequisite Programs (PRPs), systems thinking, HACCP, Good Manufacturing Practices (GMPs), Good Hygiene Practices (GHPs), food hygiene, environmental hygiene, Sanitation Standard Operations Procedures (SSOPs), Food Safety Management System (FSMS), food safety plan, food safety culture, foodborne illness outbreak, *Listeria monocytogenes*, microbiological criteria, HAZOP, Ishikawa, FMEA

## Abstract

The food industry’s failure in planning and designing of and in implementing a Food Safety Management System and its foundation elements leads, in most instances, to compromised food safety and subsequent foodborne illness outbreaks. This phenomenon was noticed, worldwide, for all food processors, but with a much higher incidence in the medium- and small-sized food processing plants. Our study focuses on the importance of Food Safety Management System (FSMS), Critical Control Points Hazard Analysis (HACCP) and the Prerequisite Programs (PRPs) as the foundation of HACCP, in preventing foodborne outbreaks. For emphasis, we make use of the example of organizational food safety culture failures and the lack of managerial engagement which resulted in a multi-state listeriosis outbreak in USA. Moreover, we correlate this with microbiological criteria. Implementation of food safety management systems (ISO 22000:2018) along with incorporation of management tools such as HAZOP, FMEA, Ishikawa and Pareto have proved to be proactive in the maintenance of a positive food safety culture and prevention of cross-contamination and fraud.

## 1. Introduction

The two common causes of major food safety incidents and recalls are undeclared allergens and cross-contamination [1].

In the USA, a majority of an estimated 75 million cases of foodborne illness are reported to have resulted from poor worker hygiene practices. Astoundingly, documentation demonstrates that between 30% and 50% of persons do not wash their hands after using the restroom. The primary means to reduce cross-contamination in a food processing plant is proper employee training [2]. These good hygiene practices are elements of a Food Safety Management System’s prerequisite programs.

The World Health Organization describes prerequisite programs (PRP) as, “essential food safety practices that need to be implemented prior to and during the installation of HACCP” [3].

HACCP is the acronym for Hazard Analysis Critical Control Point. HACCP was originally developed by Pillsbury Company USA in the 1970’s (for NASA National Aeronautics and Space Administration). The very well-known principles of HACCP refer to conduction of hazard analysis, determination of critical control points (CCP), establishment of critical limit(s), monitoring control of the CCP, establishment of the corrective action to be taken when monitoring indicates that a particular CCP is not under control, verification to confirm that the HACCP system is working effectively and documentation concerning all procedures and records appropriate to these principles and their application The circulation of HACCP around the world as the foremost system of food safety management was substantially due to the Codex report of 1997 [4].

Codex, General Principles of Food Hygiene defines Food Hygiene as, “All conditions and measures necessary to ensure the safety and suitability of food at all stages of the food chain; Properly applied prerequisite programmes, including Good Hygiene Practices (GHPs), Good Agricultural Practices (GAPs) and Good Manufacturing Practices (GMPs), along with training and traceability, should provide the foundations for an effective HACCP system [4].

An increase in food safety legislative demands is the result of widespread food scares related to microbiological hazards (e.g., *Salmonella, E. coli*), contaminants (e.g., dioxins) and animal disease (e.g., BSE) [5]. In sync, the complexity and globalization of food supply chains increased [6]. Via the robust implementation of various risk-based food safety management systems, food organizations are progressively mitigating and responding to active and risk-managed food safety hazard activities (FSMSs) [7].

In the last 20 years, a profusion of third-party food safety certification schemes (non-regulatory) has arisen which include public-based FSMSs (International Organization for Standardization (ISO) 9001:2015, Hazard Analysis Critical Control Point (HACCP) and ISO 22000:2018), and industry-based FSMSs (GlobalGAP, British Retail Consortium (BRC), Safe Quality Food (SQF), International Food Standard (IFS) and Food Safety System Certification (FSSC 22000). Notwithstanding, the implementation of these FSMSs has not met the varied expectations and challenges over the years, systems and global supply chains [8,9].

Investigation and research into FSMSs continues with regard to effectiveness and low uptake [6].

The determinants of market-based food safety management systems (FSMSs) implementation in the Middle Eastern context across Lebanon have been analyzed by Kifle-Abebe et al. [10]. They found that none of the food processors implemented industry-based FSMSs however, implementation of ISO 22000 (50%), HACCP (40%) and ISO 9001 (25.5%) took place. The key drivers for the increased implementation of market-based FSMSs include economic incentives (market orientation) and firm-specific factors such as organizational readiness, product/process characteristics, company size, and ownership structure.

The development of new, improved standards along with regulations to achieve more and more safe food is imperative and should act as pushing force [11]. Voluntary rules have been taken forward by many countries. In this context, food safety systems are interrelated with safety, quality, efficiency, reliability, interchangeability, and environmental friendliness coupled with economic factors [12].

In September 2005, ISO incorporated Hazard Analysis and Critical Control Point (HACCP) principles for food safety into the ISO 22000 quality management system. In this way there has been an integration of HACCP program and principles to quality management systems and prerequisite programs (PRPs) for the improvement of the quality and safety of the food chain in the food industry.

Two ISO technical committees have been formed, ISO/TC34 which developed ISO 22000 family for food products and management systems and subcommittee TC34/SC17 which developed systems for food safety [13] (ISO). Any party involved in the food chain business directly or indirectly can implement the standard. Now the new ISO 22000:2018 has been running.

ISO 22000:2018 provides a dynamic control method that incorporates recognized key elements, including interactive communication, systems management, prerequisite programs (PRPs) and hazard analysis and critical control points (HACCPs). Various clauses of ISO 22000:2018 are applied to the seven principles and 12 application steps of the HACCP methodology, hence their close relationship. The food safety plan–do–check–act (PDCA) cycle is one of two PDCA cycles and can be found in Operations described in Chapter 8 of the standard. Planning, implementation, regulation, correction, maintenance and updating of the PDCA cycle are described and outlined in the standard [14].

According to ISO 22000:2018, which was introduced worldwide on 19 June 2018, organizations must conduct hazard analysis to identify significant hazards [15]. CCPs and prerequisite programs (PRPs) were subsequently not limited to the sole use of the decision tree based on 4 questions. ISO 22000 imparted no detailed PRP-related information and hence was not recognized by the Global Food Safety Initiative as a standardized reference for food manufacturers in the past.

Hence, the revised version of that guideline, ISO 22000:2018, was made more effective. For example, first step is to identify the hazards and analyze them and then determine if they are a PRP, OPRP or a CCP for the significant hazards. Then the HACCP plan be put into action in terms of monitoring, control, and verification. In food industries, this step was considered to be the most critical, and also agrees well with the first principle of CODEX HACCP and ISO 22000:2018 (Section 8.5.2), which calls for the execution of hazard analysis.

Chen et al. [16] focused on significant hazard analysis, the determination and establishment of prerequisite programs, and the role of critical control point (CCP) based on ISO 22000:2018 in the catering industry. They provided guidelines and practical experiences related to the incorporation of significant hazard analysis and the use of the CCP decision tree approach to determine and update the possible CCPs of seven primary food processes.

It is well known that food chain includes organizations that are either directly involved with food or not directly involved with food processing and come in contact with either food or food ingredients [17].

The role of the new standard ISO 22000:2018 is to ensure safe food supply throughout the chain and provide a framework of internationally harmonized system.

This standard allows international trading by assuring about reliability, food quality and food safety.

The role of Food Safety Management System (FSMS) in implementation of food safety is reviewed by Panghal et al. [18]. Global Food Safety Initiative’s theme has announced the goal “once certified, accepted worldwide” in order to help industries and researchers.

Chen et al. [19], developed a food safety management system for a chaga mushroom biotechnology product manufacturer, to meet the quality demands of customers and enhance the manufacturer’s reputation. The study focused on the identification of the potentially significant hazards present at each stage of the production process for chaga mushroom capsule products, and on the assurance that the biotechnology company in question has fully implemented ISO 22000:2018 and the HACCP methodology.

The aim of this review paper is to show the paramount importance of food safety management systems and their reliance on prerequisite programs for the implementation of effective preventive controls. Even though this may seem to be a very basic principle to practice, there have been incidents that this has been neglected which consequently led to cross-contamination and foodborne illness. The case study in this document is one such incident. Moreover, the implementation of management tools such as HAZOP, FMEA, Ishikawa and Pareto will be described. Finally, correlation with microbiological criteria is discussed in the context of EU regulation 2073/2005.

## 2. Identified Barriers to Implementing Food Safety Management Systems FSMS

Lack of prerequisite programs is the #1 ranking identified barrier at 92.2%. Lack of knowledge about HACCP is the #2 ranking identified barrier at 83.5% [20]. Prerequisite GHPs must be in place first, before an effective FSMs, hazard analysis and critical control point HACCP can be implemented [21]. Nowadays, PRPs include environmental criteria and operational procedures. PRPs are encompassing the entire FSMS now, not only operational GHPs as in the past.

PRPs are recognized to be an established foundation for the implementation of HACCP. Meanwhile, HACCP and PRPs have gone unacknowledged as interdependent. For example, they are often taught separately, with more emphasis on HACCP training and certification than on PRPs. Case in hand, the ISO 22000:2005 on FSMSs contained minuscule guidance on PRP requirements that a supplemental specification for the food industry [22] on PRPs was developed for it to be benchmarked and approved by the Global Food Safety Initiative (GFSI) [23]. Thus, the symbiotic significance of PRPs to the manufacture of safe food is predominantly apparent. Recalls associated with pathogens such as *Listeria* or *Salmonella* are more often caused by PRP failure (post-process cross-contamination and | or unsanitary production environments) rather than the failure or mismanagement of critical control points (CCPs) in a HACCP plan [24].

The potential for food and beverages cross-contamination may occur at any and all food processing steps including transportation from the farm fields to processing facilities, processing steps in the food manufacturing facilities (due to deficient SSOPs, lack of personnel training, deficient GHPs, deficient good process practices (GPP), food packaging and storage). Foodborne illness outbreaks are the result of the likely risk of contamination of food products during processing and packaging and storage activities. Therefore, the identification of the root causes of contamination is crucial to understand the likely sources and paths of contamination of foodborne outbreaks and product recalls. Corrective actions and preventive control steps to mitigate their occurrence(s) can only be achieved by identifying the root causes [1]. Table 1 refers to global food safety incidents/recalls from 2008–2018 based on the type of identified hazards.

Foodborne illnesses are typically infectious or toxic in nature, caused by bacteria, viruses, parasites or chemical substances entering the body via contaminated food or water. Foodborne pathogens are known to cause severe diarrhea or debilitating infections, including meningitis.

Chemical contamination causes sudden onset of poisoning or long-term diseases, such as cancer. Foodborne diseases may tend to result in long-lasting disability and death. Unsafe food includes uncooked meats, fruits and vegetables contaminated with feces, and raw shellfish contaminated with marine biotoxins.

A formidable range of identifiable hazards (biological, chemical, physical) are existent throughout the food supply chain to the point of consumption (farm to fork) [1].

Table 2 refers to Foodborne Illness Hospitalizations in USA in the decade of 2010–2020 [1,26]. The USA Population growth from 2010 to 2020 was 234.6 million people to 331.4 million people [27].

*Listeria monocytogenes* (LM) is known to be one of the leading causes of death from food-borne pathogens particularly so in the vulnerable population including pregnant women, newborn babies, the aged and immuno-compromised individuals. LM still is documented to be a relentless challenge for ready-to-eat (RTE) food, cooked meat and fish products and dairy processors. LM is environmentally commonplace in the processing environment as it is able to survive and multiply in resistant and unfriendly conditions such as refrigeration/freezer temperature, and low pH and high sodium chloride (salt) concentration. Several RTE foods such as delicatessen meats, poultry products, seafood and dairy products (such as ice cream) are classified as a high-risk medium for LM because these foods are likely to be refrigerated/frozen thus providing suitable environments for LM to survive and multiply. Listeriosis outbreaks have been linked to seafood, dairy (such as ice cream), meat and vegetable products. However, in the last decade, listeriosis outbreaks have been linked to out-of-the-ordinary food mediums such as raw produce, ice cream and vegetable products [1]. Table 2 projects estimations of hospitalization from foodborne illness in the decade of 2010–2020.

Deficient recycling/waste disposal and lack of adequate cleaning of equipment and facilities cause the increase in the decomposition and growth of spoiled and contaminated food. This is called environmental hygiene. Inferior sanitary conditions in food processing areas and unhygienic food handling adds to poor food storage and transportation resulting in the sales/distribution of unhygienic/unsafe food. Consequently, adding to increased pest/insect population thereby increasing the risk of food contamination and spoilage [1].

### 2.1. Prerequisite Programs (PRPs)

**Figure 1:**
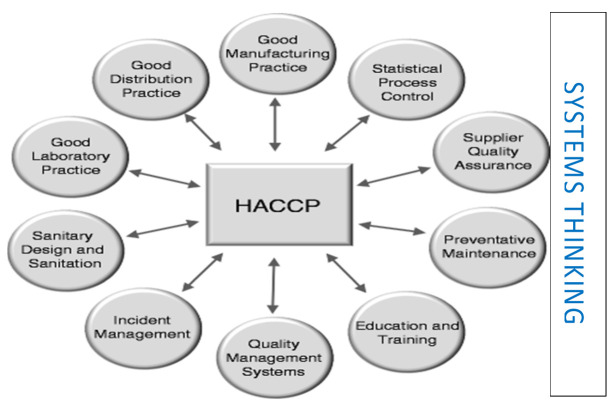
‘Prerequisite programmes (PRPs) adapted from What is a Management System? Available online: https://images.app.goo.gl/Aqez5sSfpbvjQqmK7 (accessed 20 June 2021).

PRPs provide the hygienic foundations for any food operations. The terms prerequisite programs PRPs, Good Manufacturing Practices (GMPs), Good Hygienic Practices (GHPs) and sanitary standard operating practices (SSOPs) are interchangeable in diverse parts of the globe but carry the same applicable definition (Figure 1). “Prerequisite programs” (the term) is now frequently used for systems in support of HACCP principles, all-in written and spoken languages and practices. In other words, “The concept of supporting good practices is widely accepted” [29].

**Figure 2:**
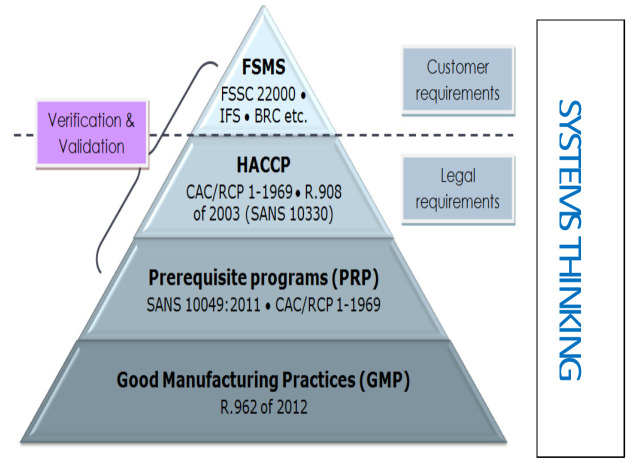
Adapted from Prerequisite Programs Ensure Food Safety Available online: https://images.app.goo.gl/jFnRtoUP3ByyLyn77 (accessed on 20 June 2021).

In Figure 2 the significant systemic importance of PRPs and HACCP is outlined. PRPs are considered the foundation|support upon which HACCP relies. “Systems thinking” can be used to help solve complex food contamination problems, and it can help deliver a structure to model solutions to urgent food safety matters. Systems thinking includes problem solving and critical thinking.

Many challenges faced today are extremely complex, and cannot be looked at from one perspective only, but they can and should be approached by looking at the system within. Looking at the system within, and having the courage to do so, restructure of the system can take place. By using systems thinking, complex problems can be solved [30]. Table 3 describes food safety competencies along with safety knowledge and skills and systems thinking.

### 2.2. Symbiotic Importance of PRPs and HACCP

(PRPs are considered the foundation support upon which HACCP relies).

**Figure 3:**
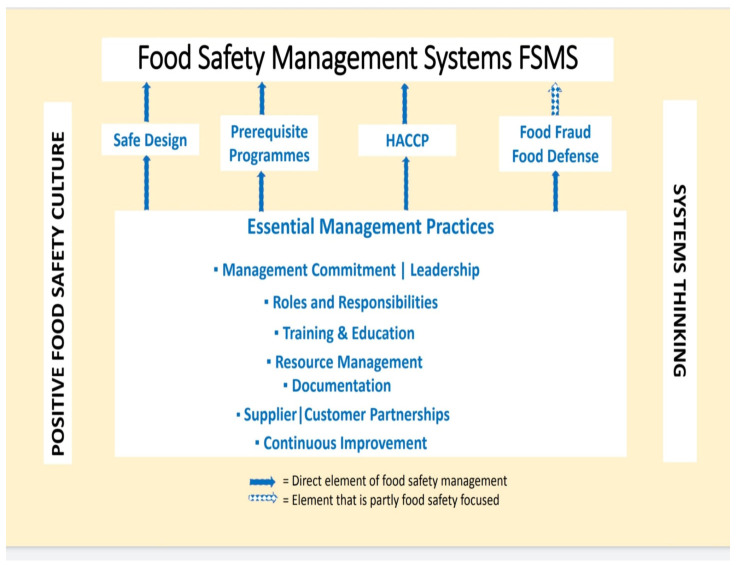
The interaction between FSMS, necessary good management practices, positive food safety culture and systems thinking Source: (Adapted from “Food Safety for the 21st Century”, Figure 9.2, page 164) [29].

PRPs are core components of the “world-class food safety program”. They have a significant support role to play as the heavy lifter foundation to other core elements. Traditionally considered as the foundational support for HACCP, we now perceive how PRPs also play an active role (in-direct) in food defense, food fraud prevention and safe food process technology and engineering. They work symbiotically with HACCP as a preventative control (PC) system. PRPs are applicable at all stages of the global food supply chain and in turn, comprise good mitigation practices for growing, harvesting, manufacturing, storage, distribution, retail, food service and cottage industry [27]. Figure 3 illustrates the interaction between FSMS, necessary good management practices, positive food safety culture and systems thinking.

#### SSOP and GMP Practices and Programs

GMP regulations guidance are designed to control the risk of food contamination with filth, dirt, scum, allergens, biofilm, chemicals, microbial particulates and other mediums during food processing.

The core GMPs for all FDA-inspected food processing sites are physical site maintenance (including outdoor detached units and portable facilities), equipment (including utensils) cleaning and sanitizing, clean equipment utensils handling and storage, pest control management, proper use and storage of cleansing solution compounds, sanitizers and pesticides, employee training, employee PPE, employee GHP, site and equipment design, and quality assurance audits.

Focused GMPs established regulations for specific food sector industries and products are in addition to the core GMPs. In other words, specific GMPs for processors: meat, poultry, seafood, dairy, feed and pet food. As such, meat and poultry processors are required to implement and maintain SSOP requirements in 9 CFR 416. USDA enforces 9 CFR 416, while FDA enforces 21 CFR 110. These particular processors are responsible for preventing contamination of their products via implementation of their documented SSOP. These processors must understand and become competent on their GMPs, because they serve as a valuable guide and tool when developing and implementing the plant’s SSOPs.

### 2.3. Current GMPs (cGMPs)

According to the Code of Federal Regulations (CFR) under Title 21 Part 110; and cGMPs in manufacturing, packing and holding human foods the following are described.

#### 2.3.1. cGMPs and Personal Hygiene

The most frequent cause of contamination is Cross-contamination of food by food handlers. Good food worker hygiene (GHP) is necessary because the cleanliness and behavior of workers determine the risk level (low risk or high risk) of cross-contamination from worker to food products and food contact surfaces. Trained, clean, sanitary, good hygiene-minded workers are of the utmost importance to produce safe food.

Several cGMPs concentrate on reducing food handler contamination. View “Code of Federal Regulations (CFR) Title 21, Part 110.10” (http://a257.g.akamaitech.net/7/257/2422/10apr20061500/edocket.access.gpo.gov/cfr_ (accessed on 20 July 2021)) [32].

Food processors of all sizes are to instruct proper handwashing to each employee by describing, in detail, how much soap they should use at what water temperature, and the proper lathering time (approx. 20 s). The processor’s employee training program documents and records all training materials. The GMPs on handwashing and facilities—such as sinks, toilets and towel racks—are presented in the SSOP format.

Finally proper maintenance of sanitary facilities and adequate supplies must be carried out including adequate quantities of soap, disinfectant, fully operational sinks supplying potable water, paper towels, toilet paper, paper seat covers, etc.

#### 2.3.2. Employer Top Management Responsibilities

Employer must provide all adequate resources to comply with and implement the above listed best practices, including top management food safety leadership engagement, sufficient labor force, training, maintenance, supplies, necessary food safety consulting, services, etc.

### 2.4. Compliance with cGMP Regulations

Here is an example of a clearly defined compliance/evaluation-easy regulation: “no pests shall be allowed in any area of the food plant”. When an inspector witnesses a mouse, or evidence of a pest (such as rodent droppings) on the site, then this is a clear regulatory violation. In contrast, other GMPs express terms as such: “clean as frequently as necessary to protect against the contamination of the food.” While some GMPs may use the terms “adequately” or “sufficient”. This unclear regulatory language is subjective. USDA-FSIS has developed more conventional requirements. Their SSOPs require processors to document adequate SSOPs and GHPs to ensure that foods are produced safely in sanitary-hygienic conditions. Whenever there is a modification, replacement or upgrade of sanitizers, cleaning agents, equipment, technologies, methods or practices, these updates and modifications should be documented and accompanied with appropriate validation.

### 2.5. Sanitation Standard Operating Procedures (SSOPs)

SSOPs are written in order to ensure sanitary conditions in food facilities. The SSOP procedures are specific to a plant but also may be similar to plants of the same or similar food sector categories. All SSOP procedures must be adequately documented and validated. When the facility’s SSOPs are in a developmental stage cGMPs could assist as a guide to the food facility.

Pre-operational “pre-op” (before daily processing begins) and operational (during processing) SSOPs include sanitation requirements to prevent direct product contamination or adulteration. The instructions on the cleaning frequency of the processing line should be addressed in the facility’s SSOPs and supporting documentation.

#### 2.5.1. Pre-Operational SSOPs (Pre-Op)

Pre-Op SSOPs include the cleaning of product contact surfaces of facilities, equipment and utensils to prevent direct product contamination or adulteration. These may include:

“(1) equipment disassembly and reassembly after cleaning, use of acceptable chemicals according to label direction, cleaning utensils, cleaning equipment and cleaning techniques. (2) instructions, and concentrations for sanitizers applied to product contact surfaces after cleaning.” [2].

#### 2.5.2. Operational SSOPs

Operational SSOPs were established to describe the daily routine sanitary procedures to be performed during operations for the prevention of direct and in-direct product contamination adulteration. Such established operational sanitation procedures must result in a clean sanitary hygienic “environment for the preparation, storage, or handling of any meat or poultry food product”. These operational SSOPs may include: (1) Equipment and utensil cleaning/sanitizing/disinfecting “during production, as appropriate, at breaks, between shifts and at mid-shift cleanup”. (2) worker hygiene procedures for the cleanliness of personal protective equipment (PPE) of outer garments, masks, respirators, Meat and poultry operations’ SSOPs and other prioritized food sector facilities where foodborne illness outbreaks originated must comply with the following regulatory requirements:

1. The facility shall have documented SSOPs detailing its daily procedures (both the pre-ops and during-ops) spelling out the steps to prevent direct in-direct product contamination adulteration. At least, these procedures shall focus on the cleaning of food contact surfaces, equipment and utensils. “The SSOPs shall indicate the frequency at which each procedure will be verified”.
2. The SSOPs are to be signed and dated by plant management or plant owner. SSOPs shall be reviewed periodically as required.
3. The facility shall designate qualified individual(s) (QI) goggles, gloves, hair restraints, handwashing, etc.
4. Special food handling in segregated raw and cooked product areas should be carried out. SSOP records shall be maintained on-site administrative storage for at least 48 h as well as maintained for a minimum of six months (appropriate off-site storage).

#### 2.5.3. Food Sector Specific SSOPs

These should be responsible for the implementation and monitoring of SSOPs and daily sanitation activities”. Maintenance of documented records of SSOP activities along with corrective actions for a minimum of six months (48 h on-site) is suggested.

#### 2.5.4. Critical Control Points

CCP is a point in a step or procedure at which a control should be applied for the prevention or elimination of a hazard or reduction to an acceptable level.

The identification of critical control points should ensure that appropriate control measures are effectively designed and implemented. In particular, if a hazard has been identified at a step where no control measure exists at that step, then the product or process should be modified at that step or at an earlier or later stage, to include a control measure. Moreover, a monitoring system per critical point should be established and implemented [33,34].

Detection of CCPs or PRPs in a food processing plant can be depicted with a 4 Q tree diagram as shown in Table 4. Receiving of raw materials is an OPRP whereas storage of raw materials, e.g., at a frozen room or refrigeration room could be a CCP in parenthesis depicting the microbiological hazard with an M.

### 2.6. CASE STUDY

Failed PRPs SSOPs GMPs GHPs—Catastrophic Foodborne Illness Case—“Blue Bell Listeriosis Outbreak of 2010–2015 and Recall of 2015”.

### 2.7. Summary

In early 2015, United States federal/state governmental public health officials formerly served notice to Blue Bell Creameries (BBC), one of the largest US makers of ice cream products, that they were responsible for a listeriosis outbreak originating back 5 years to 2010. The outbreak spread over four US states, 10 persons were sickened and four persons dead, (the Centers for Disease Control and Prevention (CDC) reported). BBC activated a weak performance of recalls intended to minimize its financial damage whilst “limiting the useful and actionable information the company provided to the nation’s consumers”. Only when confronted with extreme convincing evidence (external and internal) of its role in the outbreak did BBC recall all of its contaminated and potentially contaminated products from the US national marketplace. This case study examines the events, systemic food safety failures, and root causes that led to the foodborne listeriosis outbreak and 20 April 2015, complete-market recall wherein BBC’s top executives failed to meet “their moral responsibility to protect consumers” (by concealment, delay tactics and gross negligence).

In early 2015, BBC (a privately held company), had no food safety mission statement, no plan whatsoever to manage foodborne risks and complications | disputes, no risk-managed Food Safety Management System FSMS, no prerequisite programs, including robust Sanitation Standard Operations Procedures (SSOPs), nor product batch Laboratory Pathogen testing regimens to monitor, verify and validate their products were food safe. Additionally, no one at the operations’ managerial or top executive level was knowledgeable, trained or skilled to prepare | respond to a foodborne outbreak or a recall. After all, for 108 years, the complacent ice cream company had successfully operated without a recall.

The BBC company pled “guilty to two misdemeanor counts of shipping products across state lines” which involved the 2015 listeriosis outbreak and it “agreed to pay $19.35 million in civil charges”. The company also executed “a secret plea agreement (maybe unfavorable to Kruse) with the federal government” [36].

The 2015 *Listeria* outbreak sickened 10 people in four states and killed three in Kansas. The listeria spread from inside Blue Bell and the 18-page Grand Jury indictment charges Kruse with conspiracy and wire fraud.

“Rather than send a public notification about the contaminated products to customers and consumers,” the indictment says, “the defendant PAUL KRUSE ordered his sales employees to pull products from customers ‘shelves without disclosing the reason.” He also generated a written statement that BBC “concealed certain Blue Bell products might contain *Listeria monocytogenes*, and he directed his sales employees to give that statement to customers who asked about the removal of products” [37].

Consequently, former BBC president “Paul Kruse was charged with seven counts of wire fraud and conspiracy to commit wire fraud” on the grounds of his efforts to conceal from the public what the BBC company had already known regarding the Listeria contamination in certain BBC ice cream products. According to the indictment filed in federal court in Austin, “Texas state officials notified Blue Bell in February 2015 that two ice cream products from the company’s Brenham, Texas, factory tested positive for Listeria monocytogenes, a dangerous pathogen that can lead to serious illness or death in vulnerable populations such as pregnant women, newborns, the elderly and those with compromised immune systems”. Kruse crafted a secret action plan to “deceive certain Blue Bell customers, including by directing employees to remove potentially contaminated products from store freezers without notifying retailers or consumers about the real reason for the withdrawal”. The indictment states, “Kruse directed employees to tell customers who asked about the removal that there was an unspecified issue with a manufacturing machine”. Moreover, BBC concealed deliberately delayed the immediate recall of the BBC products and did not “issue any formal communication to inform customers about the potential *Listeria* contamination” [38].

To investigate the source of *Listeria monocytogenes* contamination, the US FDA environmental assessments were conducted at Blue Bell’s creamery facilities located in three different states: Alabama, Texas and Oklahoma.

Substantial pitfalls in HACCP-based Standard Operating Procedures (SOPs) were identified in all three production facilities as the possible contributors to *L. monocytogenes* contamination of the product (Table 5).

The most prevalent identified violations of SOPs, in all three production facilities, were related to:

(1) Failure to perform on-site, materials and product microbial testing and to take appropriate corrective actions;
(2) Failure to maintain clean and sanitized food contact surfaces to avoid cross-contamination either due to lack of performance of the used procedures for cleaning and sanitizing of equipment or due to the defective design of used equipment that does not allow proper cleaning and maintenance;
(3) Inappropriate design and construction of the production facilities to avoid product’s direct- or cross-contamination.

Additionally, in two of the production facilities, the personnel hygiene was been found being deficient and, thus, representing possible routes for product’s contamination with *L. monocytogenes* or with soil.

In only one production facility, an additional identified violation was related to the lack of monitoring the temperature—a critical control point (CCP)—a fact which favored the temperature abuse of the product.

Core FSMS Failures: Lack of managerial involvement, lack of organizational food safety culture and deceptive practices—withdrawal of products without using the recall procedure and route, and delay of recall and foodborne illness outbreak alerts.

**Figure 4:**
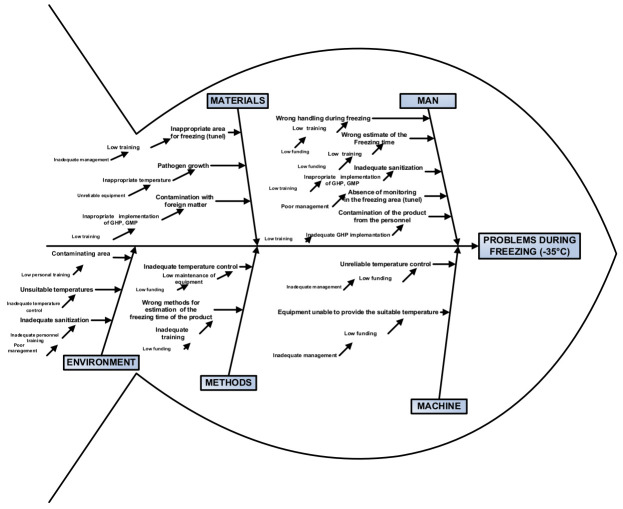

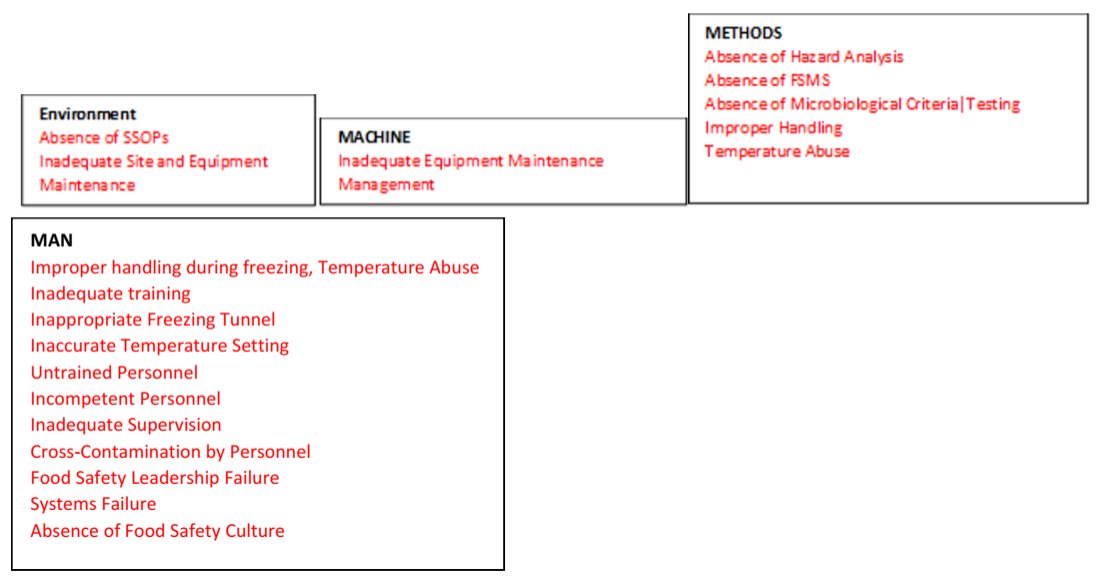
Fishbone diagram for problems during freezing.

In Figure 4 all problems related to freezing attributed to the 5 main categories (materials, man, environment, methods and machines) are described in detail. These problems along with the identified violations in the case study could act as preventative measures in the future for food companies that do not act responsibly.

## 3. Hazop

The hazard and operability study (HAZOP) [42,43,44] conducts most process hazard analysis (PHA) studies today. Proximate physical causes of accidents, also named technical or accident mechanism causes or factors should be taken into account. Causes include equipment failures, human errors by front-line personnel and external events. Chain-of-events model of accident causality is that considered for traditional PHA methods [45].

Systems theory is the one that current models of accident causality depend on. These models provide a more complete representation of the causal factors involved in accidents. PHA methods such as system-theoretic hazard analysis (STHA) should reflect these models. Another hazard analysis method is the System-theoretic process analysis (STPA) [46].

Hazop methodology also studies the effects of the deviations from design conditions.

The most common bottom-up HAZID technique is represented by HAZOP [47], with a widespread use in industry [48,49,50,51,52].

Multi-criteria Decision Making (MCDM) is a problem-solving methodology in various research fields and has shown good results in hazard analysis. Alves Viegas et al. [53] proposed and conducted a novel MCDM-based HAZOP analysis based on Strategic Options Development and Analysis (SODA), and Intuitionistic Fuzzy Sets (IFS).

Marhavilas et al. [54] developed and applied an extended HAZOP- Decision-Matrix Risk Assessment (DMRA)—Analytical Hierarchy Process (AHP) approach in process industries for identification of critical points and potential hazards and also prioritization of risks.

A case study in a sour crude oil processing-plant has been presented as a conventional HAZOP study to identify the possible fault causes of deviations in the plant.

Choi and Byeon [55] proposed HSE-HAZOP for the systematic and efficient application of health, safety and environment (HSE) engineering. They exploited the HAZOP systematic hazard analysis technique and a quantitative risk derivation method. A case study of a solution styrene butadiene rubber (SSBR) plant was used.

The new trend known as the fourth industrial revolution (Industry 4.0 represents the industrial revolution toward automation and artificial intelligence (AI)). Lim et al. [56] conducted a detailed review of the current state of the palm oil industry development toward Industry 4.0. A novel HAZOP approach is adopted to evaluate the existing problems, and identify potential implementation of Industry 4.0 technologies in the palm oil industry.

## 4. Other Management Tools in Conjunction with ISO 22000 (Fmea Analysis-Pareto and Ishikawa Diagrams)

### 4.1. Fmea

In FMEA analysis, risk of contamination and its presence at Hazardous Fraction in the final product, is expressed with the Risk Priority Number (RPN) which is defined as follows:

Where S: Severity of contamination risk, O: Occurrence of contaminated ingredient, D: Detection probability of contaminated ingredient.

RPN = S × O × D(1)

where S: severity, O: occurrence, D: detection.

Corrective action is carried out when RPN is greater than 130.

RPN assessment is carried out and corrective actions are proposed per identified hazard. Following calculation of RPN’ (after undertaking corrective actions), a new classification of Hazardous Elements is shown (Table 6, [57]).

Best expert opinion and product history (epidemiological studies) aid the analysis based on the different ways each component or subsystem might fail to comply with its intended function [58,59].

Failure mode and effect analysis (FMEA) model has been applied for the risk assessment of ready to eat vegetables manufacturing. FMEA was attempted in conjunction with cause and effect diagrams. Critical control points have been identified and implemented in Ishikawa diagrams.

Quantification of risk assessment was carried out by determining RPN per identified processing hazard. The highest RPNs (225, 225, 180 and 144, respectively) were correlated with receiving, storage and distribution, packaging and cooling and corrective actions were undertaken. Following the application of corrective actions, RPN values were below the upper acceptable limit of 130 [57].

Other case studies included FMEA analysis of salmon manufacturing. The highest RPNs (252, 240, 210, 210, 210, 210, 200, respectively) were correlated with fish receiving, casing/marking, blood removal, evisceration, filet-making cooling/freezing and distribution and corrective actions were undertaken. After application of corrective actions, new RPNs were substantially lower [60].

Another case study involved FMEA model applied in conjunction with cause-and-effect analysis for the risk assessment of octopus processing (*Octopus vulgaris*). The highest RPNs (378, 294, 280, 252, 245 and 144, respectively) were correlated with chemically contaminated product, decomposed raw materials, scombrotoxin presence in the final product, incorrectly labelled product, storage in cans (foreign matter) and defective products, and corrective actions were undertaken. Following the application of corrective actions, new RPN values led to considerably lower values (below the upper acceptable limit of 130) [61,62].

Failure Mode and Effect Analysis (FMEA) has been applied for the risk assessment of snails manufacturing. Quantification of risk assessment was carried out by determination of the RPN per identified processing hazard. The highest RPNs (280, 240, 147, 144, respectively) corresponded to sterilization of tins, bioaccumulation of heavy metals, packaging of shells and poisonous mushrooms, and corrective actions were undertaken. Following the application of corrective actions, new RPNs led to lower values (below the upper acceptable limit of 130). The application of Ishikawa diagram led to converging results thus corroborating the validity of conclusions derived from risk assessment and FMEA [63].

Varzakas and Arvanitoyannis [64] applied the FMEA model the risk assessment of corn curl manufacturing. A tentative approach of FMEA application to the snacks industry was attempted in an effort to exclude the presence of GMOs in the final product. This is of crucial importance both from the ethics and legislation aspects. Critical Control points have been identified and implemented in the cause and effect diagram and Pareto diagrams were employed towards the optimization of GMOs detection potential of FMEA.

FMEA model has been applied for the risk assessment of potato chips manufacturing by Arvanitoyannis and Varzakas [65]. Preliminary hazard analysis was used to analyze and predict the occurring failure modes in a potato processing and potato chips processing plant, based on the functions, characteristics and/or interactions of the ingredients or the processes, upon which the system depends. CCPs have been identified and implemented in the cause and effect diagram along with Pareto diagrams.

Another case study is the application of FMEA model for the risk assessment of strudel manufacturing [66]. Preliminary Hazard Analysis was employed towards analysis and prediction of the occurring failure modes in a strudel processing plant, based on the functions, characteristics and/or interactions of the ingredients or the processes, upon which the system depends. Critical control points were identified and implemented in the Cause and Effect diagram along with Pareto diagrams.

The FMEA model has been applied for the risk assessment of pastry processing [67]. The highest RPNs (225, 225 and 144, respectively, were correlated with storage of raw materials and storage of final products at −18 °C followed by freezing and corrective actions were undertaken. New RPN values led to considerably lower values. The application of Ishikawa led to converging results reflecting on the validity of conclusions derived from risk assessment and FMEA.

### 4.2. Ishikawa-Fishbone Diagrams

Ishikawa or Fishbone diagrams analyze all the hazards at all processing stages where Critical Control Points (CCPs) are incorporated. These diagrams consist of five basic axes: man, machine, materials, methods and environment. In each these axes the hazards are described in detail. Dr. Kaoru Ishikawa, a Japanese quality control statistician invented Ishikawa diagram [68]. The fishbone diagram is a systematic analysis tool looking at causes and effects at different sub levels. Due to its resemblance to fish skeleton, it is often referred to as a fishbone diagram (http://quality.enr.state.nc.us/tools/fishbone.htm) (accessed on 20 July 2021) [69].

### 4.3. Pareto

**Figure 5:**
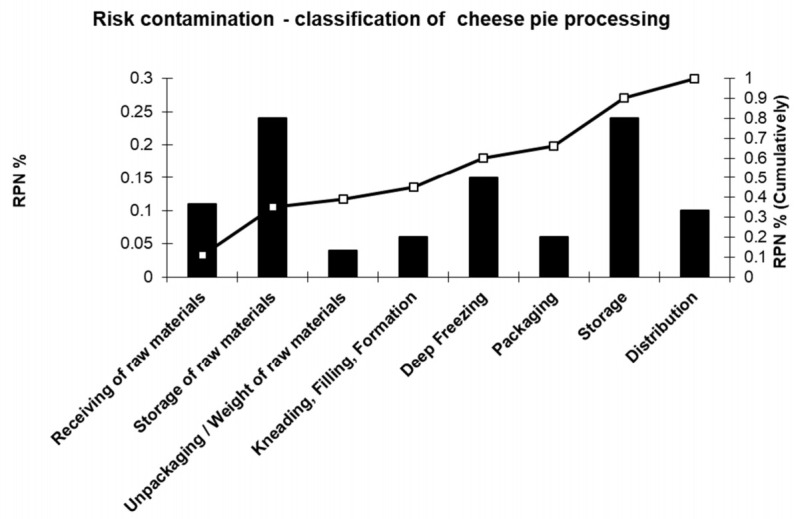
Pareto diagram of cheese pie processing prior to corrective actions.

**Figure 6:**
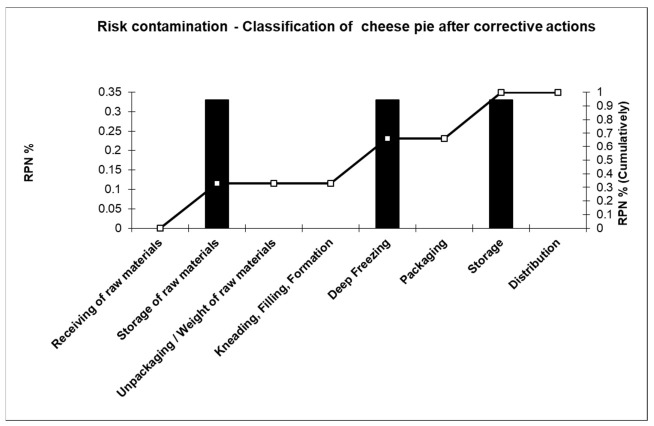
Pareto diagram of cheese pie processing after corrective actions.

A Pareto diagram is usually constructed, to determine and display high-risk processing steps and their corresponding corrective actions. Then, a second Pareto diagram is drawn focusing on cheese pie processing aiming at the determination of new risk situation (following the suggested corrective actions) (Figure 5 and Figure 6).

## 5. ‘Process-Based’ Microbiological Criteria

### 5.1. Aspects of Microbiological Criteria Related to Foods

The term «food/foodstuff» covers every unprocessed, semi-processed and processed material which is meant to be used for human nutrition. It also includes any ingredient incorporated into the food and drink, as well as any substance that comes in direct contact with them in the chain of production from start to finish (from producer to consumer). This includes all the raw materials, ingredients, means of processing, packaging and all surfaces in contact with the food or drink throughout the production process.

In recent decades, the issue of food quality and safety has become one of the most fundamental subjects of public debate in global markets and especially in the agricultural and food production industries. This debate has been fueled by many factors, but mainly due to a series of high profile scandals in the food industry, which have increased concerns on the part of consumers regarding food characteristics, production methods and their effects on human health [70,71,72,73]. Consequently, quality foods are becoming increasingly sought after by consumers globally.

In an effort to become more competitive, food producers are adopting quality and safety systems, either voluntarily or required by law. This is carried out not only so as to ensure health and safety for their products, but also to prevent customer complaints and to build and maintain customer trust/loyalty. In order to apply a given quality system to all stages of the food chain, quality management has a vital role to play [74].

However, the question as to what exactly is a quality food cannot be answered as easily as one might think, and the vagueness in the definitions of what constitutes quality has been noted by researchers such as Deming: «The difficulty in defining quality is in translating the future needs of the user into quantifiable characteristics, in a way which enables a product to be designed so as to provide satisfaction to the consumer at the price they will pay» [75].

Generally, there is agreement among academics and researchers that there are two main aspects to food quality; Objective and subjective. The former regards the physical features of a product and is related to quality control and food technology. The latter reflects the evaluation and judgments of consumers regarding perceived product quality characteristics. In any case, while food quality is an inherently complex and multifaceted concept it definitely has food safety at its core. Being the most essential variable for food quality, food safety is regulated by state legislation, in order to ensure that consumers purchase food products that fulfill their expectations regarding safety. In order to implement food quality and safety controls it is necessary to have real-time monitoring at critical points in the process. Rapid and precise methods of analysis are vital to guarantee product quality and safety, as well as compliance with labeling. The fast detection of spoilage agents such as bacteria, pathogens and other microbial contaminants in food production and processing is necessary, to reduce spoilage and secure a safe supply of food [76,77].

The food we consume is not sterile but carries a microbial load which differs from product to product due to the fact that it originates from plant and animal sources. The end microbial load of the food we consume is the sum of the natural microflora of its raw material and the microorganisms which are introduced during its harvest, processing, storage and distribution. Food related illnesses and spoilage stem from a failure to control microorganisms, in one or more stages of food production. The consequences of a mass case of food poisoning or a batch of spoiled food product can be quite serious not only for producers and sellers but also for consumers and regulatory authorities. It is therefore easy to conclude that we need a way to assess and determine the microbial quality of food in order to prevent illness caused by food unfit for human consumption, whether caused by introducing pathogens or by undesirable spoilage due to the influence of its natural microflora.

These are Microbiological Criteria which provide the guidelines we need regarding what is acceptable and safe in food and its production. These criteria determine the acceptability of a product, production lot or process based on the absence, presence or number of pathogens or/and on the quantity of toxins (or metabolites) per unit of mass, volume, surface or per batch.

Monitoring/controlling microorganisms in a food requires constant microbiological testing of samples from various stages of its production. The evaluation of this testing is made according to the predetermined Microbiological Criteria which set the acceptable range of values to which the microbiological parameters must comply. Different Microbiological Criteria are applied in each stage of production in order to ensure food is safe and will retain suitable quality to the end of its shelf life.

Given the above, food microbiology is a scientific field which, today more than ever, is essential in the effort to guarantee the efficient monitoring of microbiological parameters in the «new type» of food products being produced today. Products which undergo minimal processing while simultaneously having maximum shelf life. Microbiological analysis is the most valuable scientific tool of food microbiology in this effort and its efficiency can be optimized when used in conjunction with quality control/hazard analysis systems (ISO/HACCP).

The determination and development of Microbiological Criteria initially came about through consultation with the WHO/FAO [78] and continued to evolve through many revisions, following the contribution of the International Commission on Microbiological Specifications for Foods (ICMSF) [79]. Significant effort and funds are required for the development of effective Microbiological Criteria for a food or ingredient. Therefore Microbiological Criteria must be developed and put in place only when it is necessary and can prove effective and practical. In reality Microbiological Criteria have been set for specific bio hazards in specific food products by regulation 2073/2005 of the ICMSF [80]. Generally, Microbiological Criteria must be able to assess (1) the microbial quality of a food; (2) compliance to GHP (Good Hygienic Practices); (3) the suitability of a food or ingredient for a specific purpose and (4) the acceptance of an imported product originating from a country or region where production conditions are unknown or uncertain.

Microbiological criteria for lot acceptance determinants fall into three categories based on regulatory consequences [81,82]: (1) microbiological standard, a compulsory criterion incorporated into law or regulation; (2) a microbiological guideline, an advisory criterion to inform food operators of the microbial content that is expected in a food when best practices are applied; and (3) microbiological specifications, part of a purchasing agreement between a buyer and a supplier of a food. The state puts in place microbiological standards only when they are seen as necessary to guarantee the safety of food products subject to government regulatory control. Therefore, government bodies controlling food/food production manage the risk involved and through risk evaluation, may conclude that a microbiological criterion is necessary for a food, at different places in the food chain. Standards need to be based on tolerable level of risk and FSO for the biological hazard in question. Different types of microbiological testing (e.g., within lot, process control, investigational) may be used by industry and government). Lot microbiological testing represents one of the most common types, which compares a given microbial hazard detected in a food, to a pre-determined safety limit/range, i.e., a microbiological criterion. Microbiological Criteria can be classified into two categories, according to EU regulation 2073/2005, as modified and in effect today, as well as relevant Guidelines issued by the European Commission, regarding official microbiological sampling and testing [83]. These are «food safety» and «production process hygiene», and have the following features:

▪ Food safety standards/criteria determine lot acceptance and apply only for products for sale in the market (including finished products in storage facilities and during distribution and sale, according to Section 8, article 3 of Regulation 178/2002) [84]. These apply not only to products produced within the EU, but also to those imported to EU markets from third countries.
▪ Production process hygiene standards/criteria are an indication that a production process is functioning within acceptable limits. They are applied to specific phases of production or at its very end and contribute to the assessment of production processes, mainly for internal use by company supervisors. They are not applied to finished products already on the market and as such are not applicable to products exported within the EU or imported to EU markets from third countries.

When an official regulatory authority wishes to assess the acceptability of a product it can sample, analyze and compare the results to predetermined acceptable ranges, as stated in relevant legislation. If the sampling and analysis concerns Production process hygiene standards/criteria, then in the case of results outside normal ranges, the regulatory body focuses its actions on investigating the cause of this failure and on evaluating the impact of this discrepancy on the finished product. In any case noncompliance to production process hygiene standards/criteria is considered a failure in food safety management processes in general.

Finally, regulation (ΕU) 2073/2005 {14} applies but official regulatory authorities reserve the right to conduct sampling and microbiological analysis for microorganisms, toxins or metabolites not mentioned in said regulation. This may be deemed necessary when there is suspicion of unsafe food products in the context of hazard analysis (article 14 Reg. (ΕU) 178/2002) [84].

### 5.2. Issues Concerning Microbiological Sampling and Testing

For every category of food, the following implements, which are part of Microbiological criteria, must be determined:

- The microorganisms for which it should be tested.
- The sampling plan (number of units, frequency of testing etc.).
- Acceptable limits/ranges for every unit tested.
- The standard benchmark method of analysis to be used.
- The phase at which the criterion is applied (e.g., end product or any production stage where maximum microbial levels are expected).
- Measures in case of unsatisfactory results.

### 5.3. Results

When testing results are not satisfactory for whichever type of criteria, food producers must apply the measures determined by the regulation. If there is a tendency towards repeated unsatisfactory results, food production companies must immediately take steps to prevent the occurrence of microbial hazards.

### 5.4. Sampling Frequency

Minimum sampling frequency for products during official inspection should be based on risk/hazard analysis.

### 5.5. Testing and Scope of Application

Depending on the particular characteristics of each food, food producers must guarantee compliance of a given product to relevant criteria which must have been set by authorities, without fail. These criteria regard the following products (Table 7 captures the main micro-organisms, their toxins, metabolites found in selected food categories).

- Food ready for consumption (ready-made or ready to eat RTE).
- Fresh poultry meat.
- Minced meat and products containing meat.
- Meat products.
- Mechanically separated meat.
- Gelatin and collagen.
- Dairy products.
- Egg products.
- Living bivalve molluscs.
- Fisheries.
- Boiled crustaceans and molluscs.
- Chopped fruit and vegetables, ready for consumption.
- Sprouting vegetables and seeds.
- Non-pasteurized fruit and vegetable juices, ready for consumption.
- Slaughtered animals.

## 6. Discussion

The case study above highlights the critical importance of the successful implementation of a food safety management system and its reliance on prerequisite programs such as hazard analysis, SSOPs, microbiological criteria testing, management commitment and leadership, etc. BBC’s standing in the communities it operated in was similar to an untouchable “celebrity status” with its products popular throughout the USA for the past century. Outstandingly, the abovementioned food safety process failures in 2015 were not reported in the years before. This complicit behavior by BBC founders, management and workers went unchecked by presumably “star-struck” inspectors auditors. In fact, the recent whole genome sequencing (WGS) technology traced the L.M clone back to BBC’s products produced and consumed in 2010 establishes the aforementioned presumption.

Leadership is essential for positive food safety culture. Leaders must demonstrate ideal model behavior. The top damaging characteristic in food safety management is a food safety culture which nurtures fear. Amidst this “fear culture”, employees are disparaged from reporting potential incidents; they may cover up errors mistakes oversights, thereby increasing opportunities for incidents. We may presume this case study is one of toxic-negative-fear culture wherein there were no indications of any form of food safety management nor the important prerequisite programs [84].

Foodborne illness outbreak incidents, such as this case study, in the last decades have deteriorated consumers trust and have understandably generated a growing negative misperception on the enormity of food safety management systems [85].

We strongly believe that the implementation of food safety management systems along with other management tools such as Ishikawa, FMEA, Pareto, HAZOP could help the food industry to avoid the lack of good hygiene practices issues and to effectively and proactively monitor the critical control points (CCPs) and operational prerequisites (oPRPs). Training of all employees will play a catalytic factor in this direction and the adaptation of a positive food safety culture will minimize any likely risks, hazards, obstacles and constraints. These steps will be beneficial not only for the company but also for the consumers who will enjoy a safe, healthy, delicious and nutritious food.

## 7. Conclusions

In this review we have managed to describe the prerequisite programs as part of a food safety management system that need to be implemented for food safety and security reasons. This proves to be an excellent preventative management system that if applied along with other management tools such as FMEA, HAZOP, Ishikawa and Pareto can help the food industry protect its products from cross contamination, fraud, maintaining their authenticity, making them more traceable and improve the safety culture. A very good case study is explained from the US regarding a listeriosis outbreak and the violations that took place. Finally, the implementation of microbiological criteria is a prerequisite for all manufactured foods, especially those of animal origin and is well correlated with the case study and the implementation of FSMS.

## Figures and Tables

**Table 1:** Global food Safety incidents and/or recalls according to hazards from 2008–2018 [1,25].

Type of Recall	Average Percentage
Allergens (including mislabeling)	46.2
Cross-contamination (biological hazards)	40
Chemical Hazards	2.3
Physical Hazards	9.3
Others	2.1

**Table 2:** Estimations of Hospitalization from Foodborne Illness based on best available data. The average is based on the number of people become ill and later hospitalized because of the severity of illness. For instance: for 10 persons ill with *L. monocytogenes*, estimated 9 people hospitalized [1,26,28].

Pathogen	Occurrence in the Decade of 2010–2020
*Norovirus*	10
*Salmonella* spp.	15
*Clostridium perfringens*	1
*Campylobacter* spp.	10
*E. coli STEC*	27
*L. monocytogenes*	**90**

**Table 3:** Food safety competencies along with required safety knowledge and food safety skills and systems thinking approach Source: Adapted from “GFSI Food Safety Auditor Competencies Edition 1” November 2013 [31].

Task	Required Food Safety Knowledge	Required Food Safety Skills
Systems Thinking “Includes”: Problem Solving “Critical Thinking”	Knowledge of: Principles of systems thinking– Identification of issues as part of an overall system, rather than reaction to specific parts system improvement-special and common causes the relationship of quality management and productivity to food safety	Ability to: big picture thinking. Standing back and looking at the system as a whole, rather than just looking at the individual parts, explain the inter-relationship between quality management, operations, productivity and food safety Identify special and common causes

**Table 4:** Tree diagram for CCP and OPRP detection in food processing for a featured/representative production step according to decision of European Commission (2016/C 278/01) [35].

Procedure Step	Q1	Q2	Q3	Q4	Is This Step a Critical Control Point? (Yes/No)
	Do preventative control measures exist? (Yes/No)	Is the step specifically designed to eliminate or reduce the likely occurrence of hazard to an acceptable level? (Yes/No)	Could contamination with identified hazard(s) occur or could this increase to unacceptable levels? (Yes/No)	Will a subsequent step eliminate identified hazard(s) or reduce likely occurrence to acceptable levels? (Yes/No)	
Receiving of raw materials	Y	N	Y	N	OPRP
Storage of raw materials	Y	Y			CCP1 (M)

**Table 5:** Violations of HACCP-based Standard Operating Procedures (SOPs) identified by on-site FDA inspections Sources: US FDA Observation Reports on Blue Bell Creameries Facilities [39,40,41].

BBC Facility Location		
Alabama	Texas	Oklahoma
Identified Violations upon Inspections		
Did not carry out microbial testing in order to identify possible food contamination.	Foods were not manufactured under conditions and controls in order to minimize the potential for growth of microorganisms.”	Did not perform microbial testing to identify sanitation failures and possible food contamination.
- Protective clothing such as outer garments were not worn to protect against contamination of food and food contact surfaces. - Washing and hand sanitation was not thoroughly carried out in an adequate hand-washing facility.		Washing and hand sanitation was not properly carried out in an adequate hand-washing facility after each absence from the work station and at any time hands may have become soiled or contaminated.
Inappropriate maintainance of food contact surfaces for protection of food from contamination by any source, including unlawful indirect food additives.”	Inappropriate frequent cleaning of food-contact surfaces for protection against contamination of food.	Inappropriate storage of cleaned and sanitized portable equipment for protection of food-contact surfaces from contamination.
“The design and materials of equipment and utensils does not allow proper cleaning.”	Cleaning and sanitizing of equipment has not been shown to be effective.	Cleaning and sanitizing of equipment and utensils has not proved to be effective. Proper cleaning and maintenance cannot be carried out due to the design of equipment. Minimization of accumulation of food particles and organic matter cannot be achieved due to inappropriately smoothly bonded or well maintained seams on food contact surfaces. Thorough cleaning could not be completed due to failure to take apart equipment. Running water could not be provided at a suitable temperature for cleaning of equipment, utensils and food-packaging materials.
Preventive actions are not taken to ensure that production procedures are not affected by cross contamination.		Preventive actions are not taken to ensure that production procedures are not affected by cross contamination. Inappropriate manufacturing and packaging of foods under aseptic conditions for minimization of growth of microorganisms and contamination.
Plant construction does not prevent condensate from contamination of food -contact surfaces. Construction of Non food-contact equipment in manufacturing areas is inadequate.	Plant construction does not prevent condensate from contamination of food -contact surfaces. Inappropriate maintenance of buildings to prevent adulteration of food.	Plant construction does not prevent condensate from contamination of food-contact surfaces, and food-packaging materials.
		Inadequate manufacturing of foods at a temperature that prevents them from adulteration.

**Table 6:** FMEA Table of hazardous processing methods for food products for the receiving stage.

Defective Products											Estimated Corrective Actions Result
Production Step	Hazards	Causes	S	O	D	RPN	Corrective Actions	S’	O’	D’	RPN’
Receiving of raw materials T_r_ ≤ 5 °C Refrigeration T_r_ ≤ −18 °C freezer T_r_ = 18–22 °C Environment	Physical, Chemical or Microbiological	Wrong handling	8	5	4	160	Not required	7	2	2	28

**Table 7:** The main microorganisms, their toxins, metabolites found in selected food categories.

Category	Examples of Micro-Organisms, Parasites, Toxins and Metabolites
Raw meat: Carcasses of cattle, sheep, goats, pigs and horses	*Salmonella* spp., *E. coli*, *E. coli* O157:H7, *Yersinia enterocolitica*, *Campylobacter jejuni*, *Listeria monocytogenes*, *Clostridium botulinum* and *C. perfringens, Staphylococcus aureus* Parasites: Toxoplasma, Trichinella, Taenia and Sarcocystis Mycotoxins: Obtained from the animal through animal feed
Carcasses of broilers and turkeys	*Salmonella* spp., *E. coli*, *Campylobacter* spp., *Listeria monocytogenes*, *Clostridium botulinum* and *C. perfringens, Staphylococcus aureus*, *Bacillus cereus*
Milk and dairy products	*Salmonella* spp., *E. coli*, *E. coli* O157:H7, *Campylobacter* spp., *Listeria monocytogenes*, *Bacillus cereus*, *Staphylococcus aureus*, *Brucella* spp. Mycotoxins (aflatoxins Μ_1_ and Μ_2_), toxins from *S. aureus* and *C. botulinum* (mainly from yogurt containing fruits or nuts) shelfish
Egg products	Enterobacteriaceae (mainly *Salmonela* spp.), *L. monocytogenes*
Fishery products	*Staphylococcus* spp., *Clostridium botulinum, Vibrio* spp., *Vibrio parahaemolyticus,* histamine-producing bacteria (*Proteus morganii*), *Enterobacter* spp., *Citrobacter* spp., *Salmonella* spp., *Erysipelothrix rhusiopathiae* Parasites: *Diphyllobothrium latum*, *Clonorchis sinensis*, *Pseudoterranova* spp. Toxins: Scombrotoxin, Ciguatera and Histamine
shellfish	*E. coli*, Toxins: Amnesic shelfish toxin (ASP), Diarrhoetic shelfish toxin (DSP), Neurotoxic shellfish toxin (NSP), Paralytic shelfish toxin (PSP), Ciguatera
Vegetables, fruits and products thereof	*E. coli, Salmonella* spp., *Shigella* spp., *Listeria* spp., *Staphylococcus* spp. (mainly in mushrooms) Toxins: Mycotoxins (Patulin mainly in apples and apple juice, pears and peaches), aflatoxins (mainly in figs), toxins from *Clostridium botulinum*
Cereals and nuts	*Salmonella* spp., *B. cereus*, *S. aureus* Mycotoxins (aflatoxins)
